# Concrete Mixing Methods and Concrete Mixers: State of the Art

**DOI:** 10.6028/jres.106.016

**Published:** 2001-04-01

**Authors:** Chiara F. Ferraris

**Affiliations:** National Institute of Standards and Technology, Gaithersburg, MD 20899-8621

**Keywords:** concrete mixers, mixer efficiency

## Abstract

As for all materials, the performance of concrete is determined by its microstructure. Its microstructure is determined by its composition, its curing conditions, and also by the mixing method and mixer conditions used to process the concrete. This paper gives an overview of the various types of mixing methods and concrete mixers commercially available used by the concrete industry. There are two main types of mixers used: batch mixers and continuous mixers. Batch mixers are the most common. To determine the mixing method best suited for a specific application, factors to be considered include: location of the construction site (distance from the batching plant), the amount of concrete needed, the construction schedule (volume of concrete needed per hour), and the cost. Ultimately, the quality of the concrete produced determines its performance after placement. An important measure of the quality is the homogeneity of the material after mixing. This paper will review mixing methods in regards to the quality of the concrete produced. Some procedures used to determine the effectiveness of the mixing will be examined.

## 1. Introduction

As for all materials, the performance of concrete is determined by its microstructure. Its microstructure is determined by its composition, its curing conditions, and also by the mixing method and mixer conditions used to process the concrete. The mixing procedure includes the type of mixer, the order of introduction of the materials into the mixer, and the energy of mixing (duration and power). To control the workability or rheology of the fresh concrete, for example, it is important to control how the concrete is processed during manufacture. In this overview, the different mixers commercially available will be presented together with a review of the mixing methods. Further, the advantages and disadvantages of the different mixers and mixing methods and their application will be examined. A review of mixing methods in regards to the quality of the concrete produced and some procedures used to determine the effectiveness of mixing methods will also be given.

To determine the mixing method best suited for a specific application, factors to be considered include location of the construction site (distance from the batching plant), the amount of concrete needed, the construction schedule (volume of concrete needed per hour), and the cost. However, the main consideration is the quality of the concrete produced. This quality is determined by the performance of the concrete and by the homogeneity of the material after mixing and placement. There should be a methodology to determine the quality of the concrete produced, but only few methods and only one attempt of standardization were found in the literature. The methodology to determine the quality of the concrete mixed is often referred to as the measurement of the efficiency of the mixer. The efficiency parameters of a mixer are affected by the order in which the various constituents of the concrete are introduced into the mixer, the type of mixer, and the mixing energy (power and duration) used.

## 2. Hardware: the Mixers

There are two main categories of mixer: batch mixers and continuous mixers. The first type of mixer produces concrete one batch at a time, while the second type produces concrete at a constant rate. The first type needs to be emptied completely after each mixing cycle, cleaned (if possible), and reloaded with the materials for the next batch of concrete. In the second type, the constituents are continuously entered at one end as the fresh concrete exits the other end. The various designs of each type of mixer will now be discussed.

### 2.1 Batch Mixers

Two main types of batch mixer can be distinguished by the orientation of the axis of rotation: horizontal or inclined (drum mixers) or vertical (pan mixers). The drum mixers have a drum, with fixed blades, rotating around its axis, while the pan mixers may have either the blades or the pan rotating around the axis.

#### 2.1.1 Drum Mixers

**Fig. 1:**
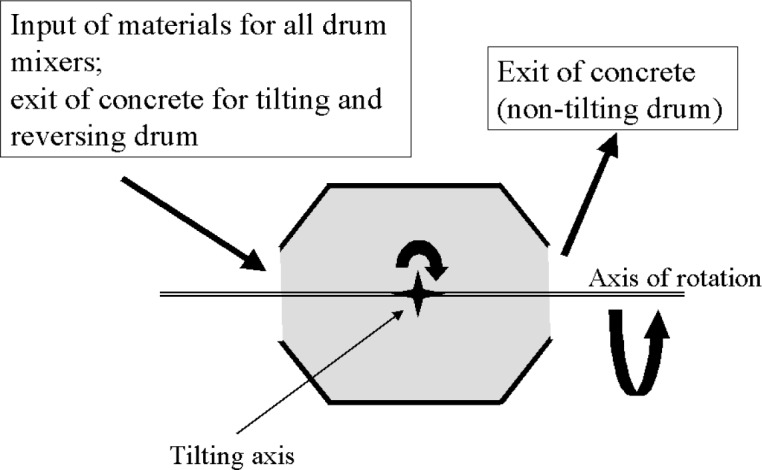
Cross section of drum mixers.

All the drum mixers have a container with a cross section similar to that shown in Fig. 1. The blades are attached to the inside of the movable drum. Their main purpose is to lift the materials as the drum rotates. In each rotation, the lifted material drops back into the mixer at the bottom of the drum and the cycle starts again. Parameters that can be controlled are the rotation speed of the drum and, in certain mixers, the angle of inclination of the rotation axis. There are three main types of drum mixers:

- non-tilting drum;
- reversing drum;
- tilting drum.

**Fig. 2:**
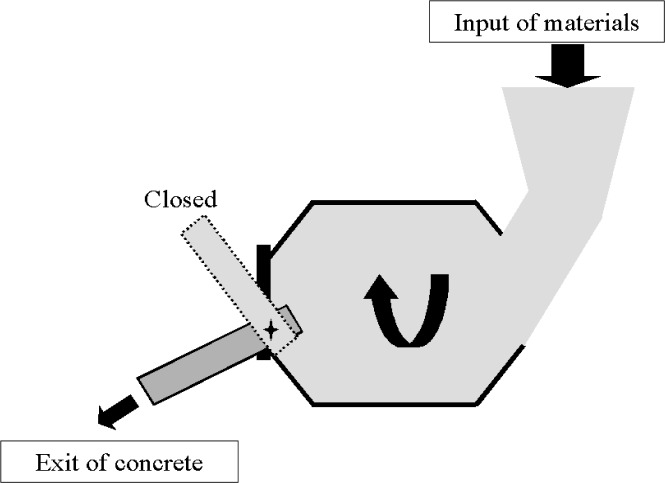
Cross section of a non-tilting mixer [1].

The non-tilting drum mixer implies that the orientation of the drum is fixed. The materials are added at one end and discharged at the other (Fig. 2).

The reversing drum (Fig. 2) is similar to the non-tilting mixer except that the same opening is used to add the constituents and to discharge concrete. The drum rotates in one direction for mixing and in the opposite direction for discharging the concrete. There are two types of blades attached to the inner walls of the drum. One set drags the concrete upwards and toward the center of the mixer when the drum rotates in one direction; the second set of blades pushes the concrete toward the opening when the drum rotates in the other direction. The blades have a spiral arrangement to obtain the desired effect for discharge and mixing. Reversing drum mixers are usually used for batches up to 1 m^3^ [1].

The truck mixers belong to the reversing category of drum mixers. The driver of the truck can control the speed of rotation with a clutch in the cabin. The speed depends on whether the concrete has been well mixed prior to being placed in the truck or whether the truck has to do most of the mixing. Typically the speed for mixing is 1.57 rad/s (15 rpm), while the transport of pre-mixed concrete uses only 0.2 rad/s (2 rpm) to 0.6 rad/s (6 rpm) [1]. In the United States, most ready-mixed concrete is mixed in trucks [2] and not pre-mixed in a plant.

**Fig. 3:**
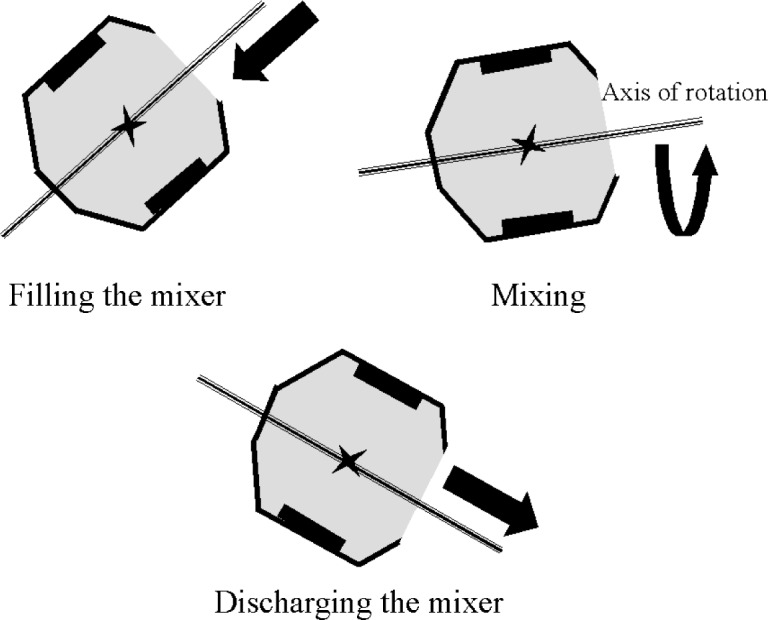
Cross section of a tilting mixer.

In a tilting drum mixer (Fig. 3), the inclination can be varied. When the drum is almost horizontal (inclination ≈ 0°), more energy is provided to the concrete because more concrete is lifted to the full diameter of the drum before dropping. It is during the drop that the concrete is knitted and mixed. Therefore, the higher the drop, the higher the energy imparted to the concrete. If the axis of rotation is almost vertical the blades cannot lift the concrete and the concrete is not well mixed. The drum axis usually stays at an angle of about 15° from horizontal during mixing. To discharge the concrete the drum is tilted downwards (Fig. 3) below the horizontal plane. The tilting drum is the most common type of drum mixer for small batches (less than 0.5 m^3^) both in the laboratory and in the field [1].

#### 2.1.2 Pan Mixers

**Fig 4:**
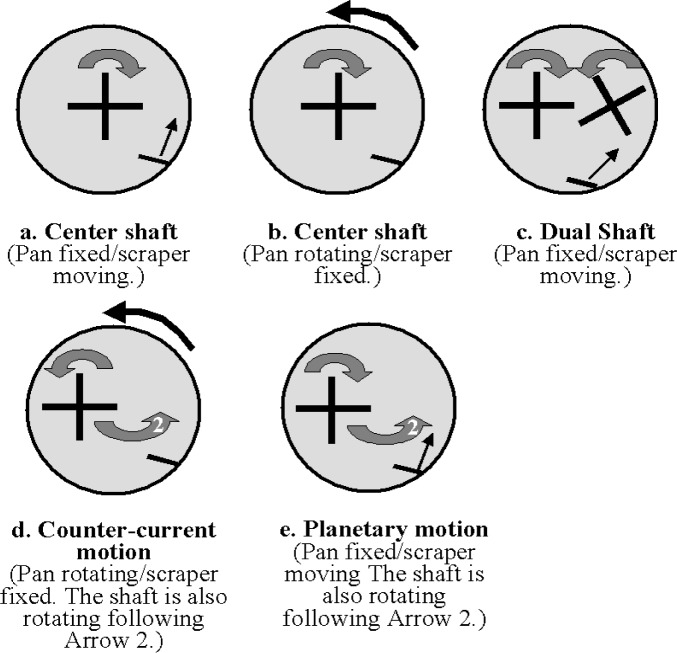
Various configurations for pan mixers. The arrows indicate the direction of rotation of the pan, blades, and scraper.

All pan mixers work on basically the same principle [3]: a cylindrical pan (fixed or rotating) contains the concrete to be mixed, while one or two sets of blades rotate inside the pan to mix the materials and a blade scrapes the wall of the pan. The shapes of the blades and the axes of rotation vary. Figure 4 shows the different combinations of blade configurations and pan. The other element of the mixer is the scraper. Sometimes the axis of rotation of the blades coincides with the pan axis (single paddle mixer, Fig. 4a and b). Other pan mixers have the axis offset [planetary motion mixer and counter-current motion (Fig. 4d and e)]. In these cases (Fig. 4d and e), there are two rotations: the blades rotate around their axes and around the axis of the pan (arrow 2 in Fig. 4d and e). The other possibility is to have two shafts that rotate in a synchronized manner [dual shaft (Fig. 4c)]. This is a blade that is suspended at an angle near the inner wall of the pan. Its role is to scrape the concrete that tends to stagnate near the wall of the pan from the wall and to push it inward so that it encounters the rotating blades. If the pan is rotating, the scraper can simply be fixed, i.e., suspended near the wall of the pan and not moving. If the pan is fixed, the scraper must move to push concrete toward the blades. Usually the individual moving parts, i.e., the blades, the pan, and the scraper, are independently powered.

To discharge the mixer, the pan is usually emptied through a trap on the bottom. For small mixers (less than 20 L or 0.02 m^3^), the blades are lifted and the pan can be removed to empty the mixer.

### 2.2 Continuous Mixers

The second category of mixers is continuous mixers [4]. As the name indicates, the materials are continuously fed into the mixer at the same rate as the concrete is discharged. They are usually non-tilting drums with screw-type blades rotating in the middle of the drum. The drum is tilted downward toward the discharge opening. The mixing time is determined by the slope of the drum (usually about 15°).

These mixers are used for applications that require a short working time, long unloading time, remote sites (not suitable for ready-mix) and/or small deliveries. A major use of these types of mixers is for low slump (non flowable [5]) concretes (e.g., pavements). Due to the short mixing time, the air content is not easily controlled even with the addition of air entraining admixtures [6].

## 3. Mixing Method

In describing the mixing process, the mixer hardware is only one of several components. The mixing process also includes the loading method, the discharge method, the mixing time, and the mixing energy.

### 3.1 Loading, Mixing, and Discharging

**Fig. 5:**
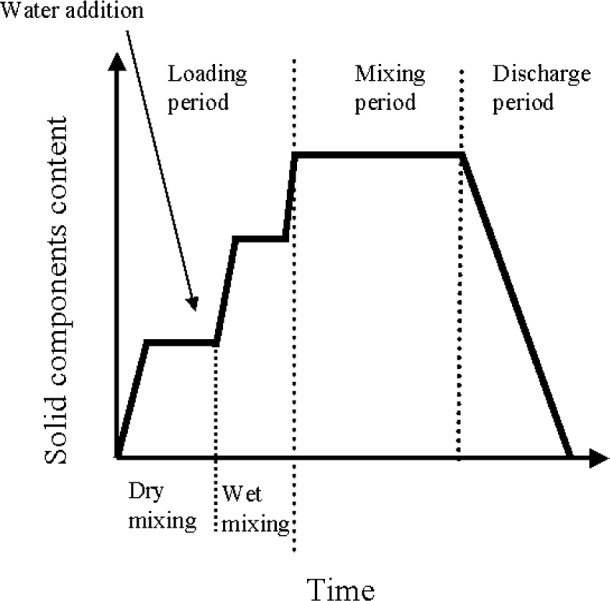
Mixing schedule [8] (see section 3.1 for further discussion of this graph).

The loading method includes the order of loading the constituents into the mixer and also the duration of the loading period. The duration of this period depends on how long the constituents are mixed dry before the addition of water and how fast the constituents are loaded. The loading period is extended from the time when the first constituent is introduced in the mixer to when all the constituents are in the mixer. RILEM (Réunion Internationale des Laboratoires d’Essais et de Recherches surles Matériaux et les constructions) [8] divides the loading period into two parts: dry mixing and wet mixing (Fig. 5). Dry mixing is the mixing that occurs during loading but before water is introduced. Wet mixing is the mixing after or while water is being introduced, but still during loading. This means that materials are introduced any time during the loading period: all before the water, all after the water, partially before and partially after.

The loading period is important because some of the concrete properties will depend on the order in which the constituents are introduced in the mixer. It is well known that the delayed addition of high range water reducer admixture (HRWRA) leads to a better dispersion of the cement. The same workability can be thus be achieved with a lower dosage of HRWRA [7]. Unfortunately, there is no systematic study, to our knowledge, that has examined the influence of the order of constituent loading on concrete properties. Most operators rely on experience and trial and error to determine the loading order of their mixer.

Very often, the mixing time is defined as the time elapsed between the loading of the first constituent to the final discharge of the concrete. RILEM [8] took another approach defining mixing time as the time between the loading of all constituents and the beginning of concrete discharge (see Fig. 5). It should be noted that solid constituents can be added at various stages of the loading period: during dry mixing, after water is added, after a second period of mixing (third slope in Fig. 5). Both definitions are acceptable. In any case, it is important that the mixing process be described fully for each batch of concrete.

The discharge from the mixer should be arranged so that it increases productivity (fast discharge), and it does not modify (slow discharge) the homogeneity of the concrete. For instance, if the discharge involves a sudden change in velocity—as in falling a long distance onto a rigid surface—there could be a separation of the constituents by size or, in other words, segregation [8].

### 3.2 Mixing Energy

The energy needed to mix a concrete batch is determined by the product of the power consumed during a mixing cycle and the duration of the cycle. It is often considered, inappropriately, a good indicator of the effectiveness of the mixer [9, 10]. The reason that it is not a good indicator is because of the high dependence of the power consumed on the type of mixture, the batch size and the loading method [11]. For example, a mixer that has a powerful motor could be used to mix less workable or higher viscosity concretes. The mixing energy could be similar to that of a less powerful mixer but one filled with a more workable concrete.

## 4. Mixer Efficiency

As it has been pointed out, the variables affecting the mixing method are numerous, not always controlled, and not a reliable indicator of the quality of the concrete produced. There is, therefore, a need for a methodology to determine the quality of the concrete produced as an intrinsic measure of the efficiency of the mixer. The concept of “mixer efficiency” is used to qualify how well a mixer can produce a uniform concrete from its constituents. RILEM [8] defines that a mixer is efficient “if it distributes all the constituents uniformly in the container without favoring one or the other”. Therefore, in evaluating mixer efficiency, properties such as segregation and aggregate grading throughout the mixture should be monitored.

### 4.1 Performance Attributes as Indicators of Efficiency

Since the macroscopic properties of concrete are affected by its composition, it is conceivable that the homogeneity of the concrete produced could be monitored by measuring the performance of specimens prepared with concrete taken from different parts of the mixer or at different times during the discharge. Properties that are often considered are

- workability of the fresh concrete as defined by the slump;
- density of the concrete;
- air content;
- compressive strength.

A disadvantage of this method is that it is indirect. It does not directly show that the concrete is homogeneous but only assumes that any potential inhomogeneity affects the properties considered. In addition, it is possible that either the measurement methods selected are not sensitive enough to local changes in composition, perhaps because the samples are too large, or that the properties selected are intrinsically not affected by inhomogeneity. The consistency in the properties is a useful guide but not a definitive indicator of product homogeneity. It can give a false sense of security about the mixing method used.

### 4.2 Composition as an Indicator of Efficiency

A more direct method to determine the efficiency of a mixer would be to measure the homogeneity of the concrete. This method does not rely on an assumption about the dependency of macroscopic properties on the concrete composition. The measure of the concrete homogeneity can be achieved by determining the distribution of the various solid constituents such as coarse and fine aggregates, mineral admixtures, and cement paste throughout the mixture. However, there are no standard tests to determine homogeneity. Nevertheless, the analysis of samples of concrete taken in various parts of a mixer or at various times during the discharge is usually accomplished by washing out the cement paste and then by sieving the aggregates. By weighing the sample before and after washing out the cement paste, the cement paste content can be estimated. The aggregates collected after the cleaning period are then dried and sieved and their size distribution is analyzed. Because the cement paste is washed out and determined as a whole, there is no provision to determine the dispersion of the mineral admixtures or very fine fillers. As demands for higher performance concretes grow, more precise methods will be needed, such as microscopic observations by scanning electron microscope (SEM), to measure the distribution of the mineral admixtures.

Based on the concept that measuring compositional homogeneity of a mixture can provide evidence of the efficiency of the mixer, RILEM [8] tried to establish a classification of mixer efficiency by defining three classes of mixers: ordinary mixer, performance mixer, and high performance mixer. Each class is defined by the range of four criteria: water/fine ratio, fine content (mainly the cement and other fine powder), coarse aggregate content (between *D*/2 and *D*, with *D* the maximum aggregate size) and air content. Several samples (the number is not specified) are taken from the mixer or from the concrete discharge, and the above parameters are measured. The average of all the measurements collected for each parameter and the standard deviation are calculated. The coefficient of variation (ratio of standard deviation to the average, *COV*) gives a measure of the homogeneity of the concrete produced, i.e., a smaller *COV* implies a more uniform mixture. Table 1 shows the criteria and the values of *COV* requested. The *COV* does not depend on the type of concrete selected because it only depends on the relative variation of the parameters for a concrete. This method, proposed by RILEM, is the only attempt by any organization to standardize the process of measuring the efficiency of a concrete mixer.

### 4.3 Hybrid: Composition and Performance as Joint Indicators of Efficiency

The hybrid method to determine the efficiency of a mixer combines the methods described in Secs. 4.1 and 4.2. The only reference to a hybrid method was found in a paper by Peterson [12], which has been adopted in Sweden. The properties selected by Peterson are

- distribution of cement content, fine aggregates and coarse aggregates in the mixer, measured as described in Sec. 4.2.;
- variations in compressive strength;
- variations in consistency as measured by the slump test with increased mixing time.

As many parameters can affect the variations in concrete performance, the method adopted by Peterson was suggested to compare mixers using the same concrete. Peterson gives three types of concrete to select from (see Table 2). These concretes were selected by him, and there were no fundamental studies to determine whether they are the optimum mixture composition for the purpose. He suggested that all three concretes be used with the mixer to be evaluated. Eight samples from each batch should be taken at various times during the concrete discharge, and the properties listed above measured. A mixer can be considered adequate if the fractional variation between measurements on any of the above properties is less than 6 % to 8 % for each batch of concrete.

### 4.4 Output Rate as an Indicator of Efficiency

Another indicator of the efficiency of specified mixer is the output rate. The output rate is the amount of concrete produced per a time interval. The output rate is not a measure of the homogeneity of the concrete produced. The output rate depends on the time needed to load the mixer, the mixing time, the discharge time, and the cleaning time, if it is a batch mixer. Very often this last stage is not considered, i.e., cleaning is not considered part of the mixing cycle. This omission is reasonable if the mixer is continuous or if it gets cleaned only once a day. Of course, for reasons of economics, the output rate should be high. However, it should be understood that it is dangerous to base the efficiency of a mixer solely on the output rate because there is no consideration of the quality of the concrete produced.

### 4.5 Mixing Energy

The mixing energy is defined as the product of the average power consumption during the whole mixing cycle and the duration of the mixing cycle. For reasons of economics, the mixing energy should be kept low but the quality of the concrete should be considered first.

Johansson [14] varied the mixing time and measured the homogeneity of the concrete discharged by measuring the variation of the composition of the concrete produced (Sec. 4.2). He determined that a longer mixing time increased the homogeneity of the concrete discharged up to a point. The curve of aggregate distribution versus duration of mixing eventually reached a plateau, implying that any further mixing would not improve the homogeneity of the concrete produced. According to the measurements performed by Johansson [14], the time at which the plateau is reached depended strongly on the type of mixer and has some dependence on the maximum coarse aggregate size. Of course, shorter mixing times that still obtain an acceptable homogeneity for a given mixture are desired. This could determine the best mixer for the application, if the loading method is kept constant. Therefore, the optimum mixing time should be determined for each concrete mixture before starting a large production.

The power consumption is often used to estimate the workability of the concrete. The theory behind this usage is based on principles of operation of a rheometer. A rheometer is an instrument that measures the stress generated by the material tested while applying a strain. In this case the strain is the constant speed of the blades and the stress is measured by the energy consumption. If it were possible to rotate the blades at different speeds and measure the power consumption at each speed, the mixer could be used to characterize the concrete’s rheological behavior. Nevertheless, while the data obtained will not allow calculation of the rheological parameters of the concrete in fundamental units because the flow of concrete in a mixer is not linear and no equations are available for such a case, the measure of the energy consumption at one speed can be used to compare concretes prepared with the same mixer [15], or to monitor the workability of a concrete while it is mixed. For a given mixture composition, if the power consumption increases, it is an indication that the concrete workability is reduced. Therefore, the operator could determine the necessity of adding more water or HRWRA to obtain the workability desired. This methodology will avoid the necessity of discharging the mixer, measuring the workability using for instance a slump cone just to determine the amount of water, or determining the HRWRA dosage needed to obtain the desired workability.

Therefore, the mixing energy is a very useful tool to determine variation in the workability of the concrete being produced. However, there is no strong evidence that mixing energy can be used to determine the efficiency of a mixer, unless the only performance requirement is the workability.

### 4.6 Wear and Tear, Cleanness

In determining mixer efficiency, the main focus has been determining the homogeneity and the quality of the concrete produced. It was assumed that the mixer was operating as designed by its manufacturer. But long usage of a mixer leads to wear of the blades and/or scraper, or the build-up of materials (hardened mortar or cement paste) on the blades, the container, and/or the scraper. Wear and build-up will change the geometry of the mixer and therefore the flow pattern of the concrete, and may lead to changes in the concrete produced [16]. To avoid this situation, the concrete mixer should be thoroughly cleaned at the end of each day of operation and the blades and/or scraper changed on a regular schedule.

It can be argued that criteria for a mixer selection should include

- ease of cleaning;
- cost and difficulty of replacing the blades or parts;
- sensitivity of the mixer to wear and tear of the blades.

## 5. Conclusions and Recommendations

Mixing is a complicated process that is affected by the type of mixer, the mixing cycle as defined by the duration, the loading method, and the energy of mixing. There are two main types of mixers: batch and continuous. In each type there are several configurations.

The efficiency of a mixer is determined by the homogeneity of the concrete produced. It could also be considered as being determined by the energy used in producing a given quantity of concrete of the required homogeneity. This homogeneity is either measured by the composition of the concrete or by the variation of the macroscopic properties, such as compressive strength and workability. It is not clear that the variation of macroscopic properties is very sensitive to variation of composition or to inhomogeneity in the concrete produced. Therefore, a direct measure of the homogeneity of the concrete produced should be the most reliable method for characterizing a mixer. A direct measurement of homogeneity relies on the determination of the concrete composition, such as distribution of the various constituents, including air content, present in various samples taken during the concrete discharge. This composition method was recommended for standardization by RILEM [8].

The mixing energy is the product of the power consumption and the duration of the mixing cycle. It is not necessarily a measure of the quality of a mixer but is used for monitoring workability during mixing, avoiding the necessity of discharging the concrete to measure slump [5].

The literature does not report problems with the mixers commercially available today. The main innovations that are currently being worked on relate to producing mixers that reduce energy consumption and the time of mixing without affecting the quality of the concrete produced.

## Figures and Tables

**Table 1:** RILEM efficiency criteria for concrete mixers [8]

Property	Performance criteria		
	Ordinary mixers (OM)	Performance mixers (PM)	High performance mixers (HPM)
*W*/*F* with *d*_f_ < 0.25 mm	*COV* < 6 %	*COV* < 5 %	*COV* < 3 %
*F* content with *d*_f_ < 0.25 mm	*COV* < 6 %	*COV* < 5 %	*COV* < 3 %
*D*/2 to *D* content	*COV* < 20 %	*COV* < 15 %	*COV* < 10 %
Air content		Δ*M* < 2 % *s* < 1 %	Δ*M* < 1 % *s* < 0.5 %

*F* is the fine-element content (units are those of mass or mass/volume)

*W* is the water content (units are those of mass or mass/volume)

Δ*M* is the maximum residual

*d*_f_ is the maximum size of the fine aggregates (mm)

*D* is the maximum size of coarse aggregates (mm)

*s* is the standard deviation.

**Table 2:** Standard concretes [12]

Concrete types	Workability	Cement content (kg/m^3^)	Aggregate max diameter and grading curve
1	Slump^b^ 100 mm to 150 mm	300	38 mm, curve 1^a^
2	Slump 20 mm to 50 mm	350	16 mm, curve 2^a^
3	Ve-Be^c^ 10 s to 20 s	350	16 mm, curve 2^a^

a Curves 1 and 2 can be found in Ref. [12].

b The slump is measured according to ASTM C143 [5].

c The Ve-Be test is measured according to Ref. [13].
